# A cell cycle centric view of tumour dormancy

**DOI:** 10.1038/s41416-023-02401-z

**Published:** 2023-08-22

**Authors:** William A. Weston, Alexis R. Barr

**Affiliations:** 1 MRC London Institute of Medical Sciences, Du Cane Road, London, W12 0NN UK; 2 Institute of Clinical Sciences, Imperial College London, Du Cane Rd, London, W12 0NN UK

**Keywords:** Cell-cycle exit, Cancer

## Abstract

Tumour dormancy and recurrent metastatic cancer remain the greatest clinical challenge for cancer patients. Dormant tumour cells can evade treatment and detection, while retaining proliferative potential, often for years, before relapsing to tumour outgrowth. Cellular quiescence is one mechanism that promotes and maintains tumour dormancy due to its central role in reducing proliferation, elevating cyto-protective mechanisms, and retaining proliferative potential. Quiescence/proliferation decisions are dictated by intrinsic and extrinsic signals, which regulate the activity of cyclin-dependent kinases (CDKs) to modulate cell cycle gene expression. By clarifying the pathways regulating CDK activity and the signals which activate them, we can better understand how cancer cells enter, maintain, and escape from quiescence throughout the progression of dormancy and metastatic disease. Here we review how CDK activity is regulated to modulate cellular quiescence in the context of tumour dormancy and highlight the therapeutic challenges and opportunities it presents.

## Introduction

Disseminated tumour cells (DTCs) present a significant clinical challenge for cancer patients due to their ability to lay dormant for prolonged periods and metastasise to secondary sites to form more aggressive tumours [1]. Despite extensive improvements to cancer treatment, metastatic relapse remains common, with recurrence occurring in, for example, 85% of ovarian cancer patients, 30% of breast, 40% of prostate cancer, and 100% of glioblastoma patients [2–6]. Tumour dormancy describes how low- or non-proliferating DTCs evade treatment or immune clearance and survive below detectable levels, then initiate tumorigenesis at metastatic sites, sometimes years later [7]. Several mechanisms of dormancy have been proposed and can be grouped into extrinsic mechanisms, such as immune suppression or restricted blood supply, or intrinsic mechanisms, including ultra-slow cell cycling, balanced proliferation and apoptosis, or a prolonged state of quiescence [7–9]. Due to the heterogenous and dynamic nature of cancer progression, all these mechanisms are likely to contribute in some way to tumour dormancy. This review will focus on the specific mechanisms of cellular quiescence and how this may be involved more broadly in all forms of tumour dormancy.

In mammalian cells, quiescence is defined as a reversible state of cell cycle arrest in the G0/G1 phase of the cell cycle. There is significant heterogeneity among quiescent cells depending on the induction signal and cellular context but all share the common feature of retaining proliferative potential [10, 11]. The terms quiescence and dormancy, and even senescence, are often used interchangeably in the literature, which can cause confusion. Here, we use quiescence in the strictest sense - to refer to a reversible G0/G1 cell cycle arrest state. Dormancy is used here to describe the clinical phenotypes, and quiescence is only one aspect of this, as described above. The reversibility of cell cycle arrest and the reduced proliferative rate of quiescence underpins the therapy evading and tumour initiating properties of dormant tumour cells. Many attribute tumour dormancy to quiescent cancer stem cells (CSCs), however, due to the ambiguity in defining CSCs, we will consider all quiescent cancer cells to be relevant in this review [12]. Targeting quiescent tumour cells could offer a therapeutic solution to tumour dormancy, either by (i) improving fractional killing of early treatments, (ii) permanently repressing their re-entry into the cell cycle, for example by driving them into senescence, or (iii) targeted killing. To better understand the role of quiescence in tumour dormancy, it will be important to fully clarify how tumour cells enter, maintain, and exit quiescence. Importantly, quiescence is distinct from senescence which describes a permanent exit from the cell cycle [13], making it unlikely for true senescent cells to reawaken and form recurrent tumours. There are reports of reversible senescence, where cells that display markers of senescence are able to resume proliferation. This is a heated debate and we refer interested readers to an excellent review on this [14]. However, we favour the view that true senescence is an irreversible state of cell cycle arrest and that ‘reversible senescence’ represents cells not fully committed to senescence, which arises due to a paucity of markers available to truly distinguish quiescent from senescent cells and a difficulty in tracking individual cell fates over very long periods of time.

**Fig. 1:**
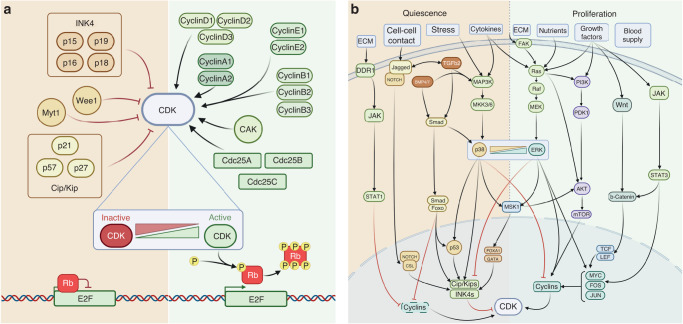
The effect of CDK regulators and the relevant signalling pathways which impinge on them. **a** CDK regulation and the subsequent effect on Rb repression of cell cycle gene expression: CDK activity is directly regulated by various activating Cyclins, Cdc25 phosphatases, and CDK activating kinase (CAK), or by repressive INK4 and Cip/Kip CDK inhibitors (CKIs) [21, 22], and Wee1 and Myt1 kinases [20]. Active Cyclin-CDK complexes promote expression of cell cycle master transcription factor E2F through hyperphosphorylation of retinoblastoma protein (Rb) [20, 27, 165]. **b** Multiple signalling pathways converge on CDK activity to determine proliferation/quiescence decisions. The proliferative and quiescent signalling pathways shown here modulate CDK activity through activating Cyclins or repressive CKIs, (outlined in (**a**)). Though the pathways here are simplified and not exhaustive of all involved, they depict those most relevant to this review and to CDK control. The ERK/p38 signalling ratio is highlighted as it is a key determinant of quiescence [30, 154, 166]. Both extracellular signal regulated kinase (ERK) and p38 are MAPK family proteins which, when activated by MAPK phosphorylation cascades, will translocate into the nucleus to regulate cellular processes and modify gene expression [167, 168]. ERK1/2 transmits growth and mitogenic signals from RAS/RAF/MEK phosphorylation cascades and stabilisation of growth factor response transcription factor families FOS, JUN and MYC which then drive cell cycle gene expression, including Cyclins^18,26–28^. Conversely, extracellular stress and inflammatory cytokines trigger phosphorylation cascades via MKK3/6 to activate p38, which reduces proliferation and promotes survival by increasing the expression of CKIs [167]. The TGFb and BMP pathway is a large pleiotropic signalling network that plays a key role in quiescence. Here we show only the relevant and simplified aspects of canonical and non-canonical pathways. Canonical TGFb signalling occurs when ligand-receptor binding causes phosphorylation of Smad proteins which translocate into the nucleus to join co-activator FoxO proteins. This active complex couples with transcription factors, such as p53, to increase expression of CKIs p15, p21 and p27. Non-canonical TGFb signalling describes when ligand-receptor binding stimulates MAPK cascades which regulate transcription via p38 mechanisms to modulate CDK activity [49, 50].

The cellular decision to proliferate or enter quiescence must be tightly regulated for healthy development and tissue homeostasis, something which is achieved through control of the cell cycle. Progression into and through the cell cycle is driven by cyclin-dependent kinases (CDKs) and their corresponding cyclins [15]. Ultimately, all quiescent or proliferative signals converge on common cell cycle regulators to modulate the activity of CDKs (Fig. 1a). If CDK activity reaches the required threshold, cells will enter the cell cycle and proliferate. If not, cells will remain in quiescence. In this way, cells can integrate intrinsic signals, such as cell size and stress, including replication stress or metabolic stress, with extrinsic cues, including ECM composition, nutrient availability, or growth factors, to ensure proliferation only occurs when required. Unsurprisingly, dysregulation of upstream pathways, or mutations in the central control mechanisms of the cell cycle are heavily associated with cancer and other pathologies [16]. Cellular quiescence requires reduced Cyclin-CDK activity, most commonly by either by upregulating CDK inhibitors (CKIs), or by degrading or downregulating cyclins (Fig. 1a).

In this review, we outline the key molecular mechanisms that converge on and impact CDK activity and, as such, regulate cellular quiescence during tumour dormancy. We provide examples of how cellular and acellular factors can impinge on these mechanisms to allow cells to induce, maintain, and exit quiescence. The models used to study tumour dormancy are largely based in vitro in cell lines and in vivo in mouse models, where cancer cells can be injected into mice and their ability to colonise and proliferate at metastatic sites is used as a measure of how dormant they are. These models both have their limitations in modelling human disease where tumours can remain dormant for decades, far beyond the available time to track cells in vitro or the maximum lifespan of 2.5 years for a mouse. However, important insights have been made in these systems and in our review, we discuss the experimental systems used and include information where these have been linked to human disease.

## Controlling quiescence/proliferation decisions through CDK activity

The cellular decision between quiescence or proliferation is thought to be made at the restriction point (RP), a time in the G1 phase of the cell cycle after which cells no longer require growth factor stimulation to proliferate [17, 18]. Growth factor stimulation in quiescence increases CyclinD expression (Fig. 1b), increasing CyclinD-CDK4/6 activity over a critical threshold. CyclinD-CDK4/6 phosphorylates the transcriptional repressor retinoblastoma protein (Rb) causing partial release of its inhibition of the transcription factor family E2F [19]. E2F transcription factors promote the expression of many proliferative genes including CyclinE, increasing CyclinE-CDK2 activity that further phosphorylates Rb. This creates a positive feedback loop through Rb hyperphosphorylation, allowing full E2F activation and ‘commitment’ to proliferation [19, 20]. Expression of CKIs of the INK4 family (p15^INK4B^, p16^INK4A^, p18^INK4C^ and p19^INK4D^) that specifically inhibit CDK4 and CDK6 [21], or of the Cip/Kip family (p21^Cip1^, p27^Kip1^ and p57^Kip2^) capable of inhibiting all Cyclin-CDK complexes [20, 22], increase the threshold of CDK activity required to leave quiescence. One key regulator that can drive increased CKI levels is p38 kinase (Fig. 1b). p38 can also directly inhibit E2F transcriptional activity by phosphorylating the N-terminal region of Rb to make it unresponsive to Cyclin-CDK activity [23–25].

The precise timing and mechanisms underpinning passing the RP are a hot topic of current investigation [26], and above we provide the ‘canonical’ view. There is also heterogeneity regarding a cell’s ‘depth’ of quiescence, a term which will be used to describe a cell’s distance from the critical threshold for CDK activity, or the RP. Experimentally, quiescence depth has been demonstrated by keeping cells in quiescence (e.g., by serum starvation) for different periods of time and then stimulating them to re-enter the cell cycle. Quiescent cells that have been out of the cell cycle for longer required more time or an increased stimulus (e.g., a higher concentration of growth factors) to re-enter the cell cycle [27].

In tumour dormancy, multiple growth factors and signals from the metastatic niche will contribute to proliferation/quiescence decisions. All these inputs are transduced and integrated through a few key signalling pathways that ultimately impact CDK regulators and CDK activity (Fig. 1b) [28]. We look at how these inputs decrease CDK activity such that cells either enter or maintain quiescence, which contributes to how tumour dormancy is established and maintained, and how CDKs can be reactivated, which can drive tumour relapse.

## Inducing dormancy: quiescence and the ‘pro-dormancy’ niche

Though previously considered to be a late stage in cancer progression, dissemination of tumour cells may occur early in tumorigenesis. The delay of detectable metastatic lesions is due to cellular dormancy induced by the non-permissive environment of metastatic sites [7]. DTCs that invade the blood or lymphatic system disseminate widely in suspension, and frequently lodge in unfamiliar tissues with alternative homeostatic mechanisms, which may favour quiescence over proliferation [9]. Mounting evidence points to the existence of various ‘pro-dormancy niches’ in which DTCs lay dormant for extended periods of time before escaping quiescence to form metastatic lesions. However, even before extravasation from circulation into a new metastatic site, DTCs can enter a dormant-like state due to their loss of cell adhesion and nutrient availability. Floating spheroids isolated from ovarian cancer patients were shown to be arrested in quiescence, with increased levels of the Rb-like protein p130, the CKI p27 and reduced CDK4/6 activity [29]. Loss of adhesion in these spheroids removes integrin-FAK/RAS/ERK signalling and initiates a stress response that causes inhibition of AKT/PI3K signalling (Fig. 1b). This subsequently pushed cells into a ERK^low^/p38^high^ profile [29], something which reduces proliferation and contributes to a quiescent phenotype [7, 25, 30–32], often through a common effector MSK1 [33, 34]. Indeed, in vitro models using aggregates of squamous carcinoma cells grown in a nutrient-deprived suspension, have been shown to arrest via growth factor-independent epidermal growth factor receptor (EGFR)-Y1086 autophosphorylation, which leads to reduced AKT signalling (Fig. 1b) and reduced CyclinD [35]. Here we describe some of the key mechanisms and niches known to induce DTC quiescence.

### Secreted factors

Once DTCs invade a tissue from the vasculature they are frequently met with non-orthotopic microenvironments which promote quiescence through cell-cell adhesions, secreted factors, and specific ECM interactions [7]. While these niches exist in the brain, lung and liver, it is perhaps best studied in the bone marrow^71,72^, therefore, this review will mainly focus on examples from this niche. Haematopoietic stem cells (HSC) are maintained in the endosteal niche within the bone microenvironment (BM) by spindle-like osteoblasts through several key secreted signals which are hijacked by DTCs to induce their quiescence and cell survival. One example is secreted Wnt5a, which has been shown to induce quiescence in the prostate cancer cell line, PC-3, in vitro [36]. Wnt5a binding its receptor ROR2 promotes SIAH1 and Ebi to ubiquitinate B-catenin for degradation, inhibiting proliferative canonical Wnt signalling [37–40] (Fig. 1b) and reducing the expression of MYC and CyclinD to induce quiescence [36–38, 40]. In vivo studies using mouse models of prostate cancer confirmed that addition of Wnt5a reduces tumour burden (prolonged dormancy), while Wnt5a knockdown leads to increased detectable metastatic foci in the bone, which appeared sooner than in wild-type mice [36, 41]. Further evidence shows that BM-derived Wnt5a is significantly reduced in aged mice, which may account for the late development of metastatic lesions [36, 42]. Moreover, high ROR2 expression correlates with improved bone metastasis-free survival in prostate cancer patients [36]. While best characterised in bone marrow, similar research has shown Wnt5a to reduce cell cycling of melanoma metastases in the lung microenvironment, accompanied by elevated p21 expression, and delayed metastatic outgrowth [43].

Similar evidence exists for secreted TGFb and BMP signalling contributing to cellular quiescence [44–46], acting through canonical [47, 48] and non-canonical [49, 50] pathways to modulate CDK activity (Fig. 1b). Studies have implicated osteoblast-derived BMP5/6/7 in the quiescence of Myeloma cell lines [51] while in vivo models of metastatic breast cancer showed that BMP secreted by lung stromal cells prolongs phenotypic dormancy [52]. A paper by Kobayashi [53] gave in vitro and in vivo evidence that BMP7 secretion in the bone was preventing the outgrowth of metastatic prostate cancer cells through upregulation of p38 and p21, which caused a reversible state of cell cycle arrest. Moreover, murine models of metastatic prostate cancer have shown osteoblast secreted TGFb2 and GDF10 are key to promoting cellular quiescence and maintaining tumour dormancy in the bone [41]. Both TGFb2 and GDF10 ligands bind TGFb3R to increase nuclear translocation of (active) phospho-p38 [41, 54], which deepens quiescence in prostate cancer cells by promoting expression of the CKI p27 [55].

### Cell-cell interactions

In addition to secreting key factors, osteoblasts hold HSCs in the niche through cell-cell interactions via N-Cadherin, bringing about increased Notch2 signalling [56, 57]. High Notch2 signalling has been shown to cause cell cycle arrest in small cell lung cancer (SCLC) cell lines by upregulating CKIs p21 and p27, and inhibitory phosphorylation of ERK1 and ERK2 (Fig. 1b) [58]. A paper by Capulli et al. [59] modelling metastatic breast cancer, showed that disseminated cells were high in N-Cadherin, allowing them to compete with HSCs to engraft in the endosteal niche, where they are then kept quiescent by Notch signalling. Moreover, silencing of Notch1 and Notch2 abrogated the dormancy phenotype in mice with increased metastatic foci in the liver and other organs suggesting increased proliferation and metastasis. The same study highlighted how breast cancer patients with Notch2^high^ cancers have improved prognosis pre-treatment as their primary tumours are less proliferative leading to smaller primary lesions, but worse than Notch^low^ patients after chemotherapy [59], potentially because of an increased number of quiescent DTCs meaning metastatic relapse is more common.

Similar research using both spontaneous and intracardial injection-based mouse models of metastatic breast cancer, showed how quiescent DTCs accumulate at the microvascular endothelium of lung, brain, and bone [60]. Using in vitro models of lung microvascular networks, Ghajar et al. [60] showed how Thrombospondin-1 (TSP1), expressed on mature endothelial cells, maintained DTC quiescence. TSP1 is a surface protein of mature endothelial cells but can also induce quiescence when secreted by BM-derived myeloid cells [61]. Quiescence is likely due to TSP1 binding CD47, which upregulates CKIs p21 and p27 expression, while simultaneously inhibiting CyclinD1 [62]. Though the exact mechanism through which this occurs remains unclear, TSP1 has also been shown to upregulate TGFb signalling to repress tumour growth in cells isolated from primary breast tumours, suggesting a possible common role for TSP1 in various dormant cancers [63].

### Cell-ECM interactions

A wealth of evidence exists showing how extracellular matrix (ECM) composition and structure can influence tumour progression, as well as contribute to the normal and cancer stem cell niche [64, 65]. Multiple in vitro studies have shown how an ECM substrate is able to slow cancer cell proliferation in comparison to plastic monolayer cultures [66, 67]. Components of the basement membrane, including Collagen 4, TSP1 and Laminin-1 are known to induce quiescence in oestrogen-receptor positive (ER+) breast cancer cells growing in vivo or in 3D models [60, 68, 69]. The basement membrane is a known player in stem cell pool maintenance, and though the exact pathways are not clear in cancer, it is likely to be a similar mechanism in DTC quiescence. ECM biomechanics are also thought to affect cancer cell behaviour and bring about quiescence. Hepatocellular carcinoma (HCC) cell lines proliferate rapidly when cultured on a ‘stiff’ matrix due to increased focal adhesions, leading to elevated FAK/ERK and PKB/Akt signalling (Fig. 1b). However, when moved to a ‘soft’ substrate, HCC cells become quiescent, exhibiting decreased levels of CyclinD and CyclinE [70]. Similar evidence exists for breast cancer cells proliferating in more ’stiff’ ECM and remaining quiescent in softer ECM, mimicking brain metastasis [71]. Indeed, increased stiffness has been associated to more aggressive tumours in pancreatic, ovarian, bladder and glioblastoma tumours [72–75], indicating its support of proliferation rather than quiescence.

Though there are myriad signalling pathways which can bring about quiescence, these appear to be elevated in ‘pro-dormancy niches’ where DTCs are more likely to lodge, enter quiescence and survive for prolonged periods. Frequently, DTCs inhabit stem cell niches due to their adaption to slow proliferating and protective mechanisms required for stem cell maintenance. Some evidence suggests DTCs specifically target these niches based on chemoattraction and cell-binding, however, there is also a strong selection pressure against more hostile environments.

## Maintaining dormancy—avoiding proliferation and death

The extended latency of quiescent cancer cells requires sustained antiproliferative signals such as those outlined above. However, there is mounting evidence that DTCs can maintain and compound their quiescent state via DNA modifications, autocrine signals, and niche modifications. Here we describe examples for quiescence maintenance mechanisms which are co-opted by cancer cells to maintain proliferative potential as well as avoid apoptosis or senescence.

### Transcriptional rewiring for quiescence

A key aspect to maintaining quiescence in healthy cells is a modified transcriptional programme which consolidates antiproliferative signals. Many anti-proliferative gene regulators, such as p53, SALL2 or MXI1, are dysregulated in cancer to allow tumour formation despite cell stress or serum starvation [76–80]. However, some regulatory transcription factors have now been shown to perpetuate tumour dormancy by promoting a quiescent gene expression profile. Overexpression of the Lymphocyte Kruppel-like factor (LKLF) is sufficient to induce and maintain quiescence in leukemic T-cells in vitro [81]. LKLF maintains quiescence through inhibition of MYC, while associated transcription factors, Tob and FOXO, consolidate this quiescence by promoting p27 and antagonising CyclinE expression [81]. Recent work from Nobre et al. [82] in a spontaneous mouse model of Her2+ breast cancer identified an upregulation of the transcription factor ZFP281 in early metastases isolated from the lung, which promoted a quiescent-like expression profile that prevented tumour outgrowth. Typically expressed in mouse and human embryonic stem cells, ZFP281 induces epithelial-to-mesenchymal transition through upregulation of Snail and Zeb1 transcription factors, which has been previously shown to suppress CyclinD1 and D2 expression [83, 84].

Chromatin modifications can produce longer lasting changes in gene expression to maintain quiescence. Histone methylation and acetylation can be activating or repressing, depending on context, and will often modify a network of related genes. Dormant human epidermoid carcinoma Hep3 cells in vitro and in mouse xenograft models were shown to enter quiescence through upregulation of DNA methyltransferase DNMT1, leading to the repression of proliferative genes [85]. The histone methyltransferase SMYD5 has also been shown to be necessary for dormancy of lung metastases in mice injected with breast cancer cells [86]. The orphan nuclear receptor NR2F1, commonly mutated in cancers [87, 88], was shown to be epigenetically upregulated downstream of TGFb/p38 signalling (Fig. 1b) in murine models of dormancy [85], as well as in DTCs isolated from long-term prostate cancer patients [89, 90]. NR2F1 and retinoic acid receptor β (RARβ) increase expression of the pluripotency factor SOX9 via activating Histone H3K4me3 modifications, resulting in increased expression of CKIs p15, p16 and p27 [89]. However, p38/NR2F1-induced quiescent cells maintained a globally repressive chromatin state with a H3K9me3^high^ and H3K27me3^high^ profile, typical of long-lived quiescent cells. Additional evidence from osteosarcoma xenografts in mice has shown the transcriptional repressor Hairy and Enhancer of Split 1 (HES1) to be associated with tumour dormancy and a more repressive chromatin state [91]. Downstream of Notch and Hedgehog signalling, HES1 contributes to preventing tumour cell differentiation by recruiting repressive histone deacetylases (HDACs) to its target genes, leading to chromatin compaction [91, 92]. This promotes quiescence and maintenance of the dormancy phenotype by repressing pro-apoptotic gene expression, or differentiation markers such as Mash1 or NeuroD [91].

The reversible nature of quiescence is heavily reliant on transcriptional changes and chromatin modification. These transient changes allow cells to maintain a non-proliferative state with low CDK activity and deepen quiescence by consolidating CKI expression, while avoiding terminal differentiation, senescence, or apoptosis. However, a strong enough mitogenic signal could still overcome these mechanisms to increase CDK activity and surpass the critical threshold for proliferation. Therefore, DTCs will often supplement their transcriptional changes with local niche modifications that further deepen quiescence.

### Niche modification for quiescence

Rather than merely surviving in existing pro-dormancy niches, DTCs can actively remodel their microenvironment to improve the survival of metastases. Typically, the deposition of ECM proteins has been associated with escape from quiescence at metastatic sites such as the perivascular niche [93]^.^ However, there is now evidence for DTCs modifying their metastatic environment to promote quiescence. This is likely to be commonplace when we consider the variety of tissues which can support the same DTCs; brain, bone, liver, and lung all support breast cancer cells [94]. An in vitro study by Barney et al. [95] showed ER+ breast cancer cells maintained in a prolonged quiescent state deposit an organised fibrillar Fibronectin (Fn) matrix to promote cell survival and maintain quiescence. Serum starved breast cancer cells received sustained autocrine TGFb2 signalling (Fig. 1b) which initiates matrix remodelling via integrin α5β1 binding and downstream Rho kinase activity [96]. The subsequent sustained αvβ3 and α5β1 integrin binding by Fn permits arrested cells to suppress apoptosis via expression of Bcl-2 [97]. Indeed, quiescent breast cancer cells were shown to re-enter the cell cycle following specific degradation of Fn, and conversely, serum stimulated recovery of quiescent cells lead to a similar degradation of the Fn ECM by secreted matrix metalloproteases (MMPs) [95]. More recent findings have shown that quiescent HNSCC lines injected into mice will deposit a collagen-3 (COL3A) rich matrix which promotes further COL3A expression via the collagen receptor DDR1 and downstream STAT1 signalling [98]. The COL3A rich matrix prolonged dormancy/reduced tumour burden in DTCs [98], potentially via STAT1 induced proteasomal degradation of CyclinD/CDK4 complexes [99].

As well as manipulating the ECM, DTCs can use local autocrine signals which ‘deepen’ their quiescent state and promote survival. Autocrine DKK1 enforces a state of quiescence in lung and breast cancer cells by inhibiting Wnt3a to reduce proliferative Wnt signalling [100], as well as reducing expression of the NK cell receptor ULBCP, allowing quiescent cells to escape immune clearance by natural killer cells. DKK3 has a similar quiescence inducing effect on prostate cancer cells in the BM, though the exact mechanism remains unclear [101]. A comparable autocrine Wnt3a inhibition mechanism was also identified in prostate cancer cells, responsible for reduced metastatic foci in mice [36]. Autocrine IGF1 signalling has recently been identified as an essential survival mechanism for quiescent cancer cells derived from murine pancreatic tumours, allowing for growth and apoptosis resistance in spite of mutations in KRAS or MYC. Insulin-like growth factor-1 (IGF1) was upregulated in the same cells, leading to increased AKT activation and cell survival. AKT signalling increased the proportion of functionally inactive apoptotic proteins BIM and BAD, and increased expression of the apoptosis inhibitor XIAP. Indeed, inhibition of IGF1/AKT signalling alongside c-MYC or KRAS inhibition increased pancreatic cancer cell clearance and delayed tumour recurrence in these mice [102].

Frequently, quiescence-inducing signals also stimulate downstream stress response pathways to promote cell survival. p38 can induce quiescence through upregulation of various endoplasmic reticulum stress response pathways to maintain cell health [25]. Quiescent human epidermoid carcinoma cell models subjected to environmental stress and chemotherapeutic insult in vitro have been shown to upregulate BiP and Eukaryotic translation initiation factor 2-alpha kinase 3 (PERK) in a p38-dependent manner, leading to increased protein chaperone production and inhibition of the apoptotic protein Bax [103]. Concurrently, p38 upregulates the survival transcription factor ATF6a when in a state of quiescence in these same cells^115^. ATF6a is trafficked from the endoplasmic reticulum to the nucleus by MKK6/p38 where it promotes the expression of unfolded protein response (UPR) genes, as well as increasing Rheb activated mTOR signalling. While AKT activated mTOR typically drives proliferation (Fig. 1b), here Rheb/mTOR promotes cell survival mechanism that allow quiescent DTCs to withstand stressors from their metastatic niche and sustain a dormant phenotype [104, 105].

There is some debate as to whether quiescence is induced and maintained through constant signalling input, or with a switch-like mechanism of cell reprogramming. Though external signals often drive cells into quiescence, the critical threshold of CDK activity can be increased further by switch-like changes to the chromatin structure and the transcriptional profile. These switches often feedback through autocrine signals, anti-apoptotic and stress pathways, and niche modifications which will combine to reduce CDK activation and consolidate quiescence. In some cases, DTCs will receive conflicting signals which may neutralise or reduce the impact of one another on CDK activity. However, provided cells have not entered senescence, even those DTCs in the deepest quiescence can re-enter proliferation, so long as the threshold of CDK activity is reached.

## Tumour relapse—quiescence to proliferation

The process by which DTCs exit quiescence and initiate metastatic tumours will, in part, involve a gradual cessation of the pro-quiescence signals depicted previously^36,108^. Moreover, cellular quiescence may also be broken by passive events such as random epigenetic drift or decay over time^109–111^. Though oncogenic mutation is slowed to almost zero in quiescent DTCs, a longitudinal in vitro study by Magnani et al. [106] suggests stochastic awakening events are inevitable due to the inherent instability of quiescent cell chromatin, which will decay with age to allow reactivation of proliferative genes. However, quiescent cell awakening can also be triggered by microenvironmental changes occurring through inflammation, ageing, growth factor stimulation, ECM remodelling, or migration to new tissues. Here we discuss some examples of quiescent cell activating signals and describe how they act to increase CDK activity.

### Vascular perfusion and growth factors

While angiogenic dormancy is distinct from cellular quiescence [107], vascular perfusion of a dormancy niche is a strong predictor for DTC awakening and metastatic outgrowth. Neo-vascular tips create highly proliferative environments, rich in growth-promoting factors, oxygen, and nutrients which can be hijacked by DTCs to exit quiescence. A surplus of growth factors supplied by the blood will most often activate MAPK/Ras/ERK pathways to induce proliferation (Fig. 1b). Though established tumours are capable of inducing angiogenesis as previously reviewed [108, 109], there is currently no evidence of quiescent cells doing the same to drive outgrowth. However, injury and tissue repair are likely to encourage angiogenesis and other forms of tissue remodelling which could activate quiescent DTCs. Ghajar et al. [60] showed the pro-dormancy protein TSP1 was downregulated in neovascular tips of mice compared to established blood vessels, while TGFb1 was upregulated and, triggered proliferation of injected breast cancer cells. TGFb1 stimulates stromal fibroblasts to deposit an ECM matrix rich in Periostin, Tenascin-C (TNC) and Fn which is optimal for proliferation [60]. TNC and Periostin are known to amplify proliferative Wnt signals, to increase downstream activity of MYC and CyclinD [30]. In addition, Fn co-assembly with TNC increases integrin signalling capacity, driving proliferation through FAK/ERK pathways (Fig. 1b) [110]. Fn substrates have also been shown to promote motility in metastatic prostate cancer cell lines in vitro [111–113], which could enable DTCs to migrate to proliferative tissues, something which is made more likely by the proximity to blood vessels.

### Inflammation and stress

Chronic inflammation is closely linked with cancer development and metastasis [114], and has been connected to relapse in patients with breast, oral and endometrial cancer [30, 115–117]. Prolonged inflammation leads to accumulation of immune cell secreted inflammatory cytokines such as interferon-gamma (IFN-y), interleukin-1/6 (IL1/IL6) and Tumour necrosis factor (TNF) which have all been associated with metastatic relapse in patient studies of various cancer types [118, 119]. However, in vitro and mouse studies have attributed a large part of this association to mechanisms involving immune-mediated dormancy [120, 121], which we will not discuss here. In the context of quiescence, IL6 has been shown to drive proliferation in breast cancer by activating JAK/STAT3 signalling, which increases expression of MYC, B-catenin and CyclinD (Fig. 1b) [122, 123]. This effect can be boosted by IFN-y activation of JAK-STAT, MAPK and PI3K signalling, which can drive proliferation as shown in Fig. 1b [124]. Khazali et al. [125] cultured breast cancer cells in an ex vivo liver system, to show how hepatic stellate cells could secrete the inflammatory cytokine IL8 to reduce cancer cell quiescence under serum starved conditions. This suggested that inflammation of the liver could contribute to late emergence of metastases in the liver. Meanwhile, immune cell secreted IL1 and TNF are highly pleiotropic cytokines that can indirectly induce exit from quiescence through activation of angiogenic factors IL6, IL8 and VEGF, or by modification of the ECM via expression of MMPs [126, 127]. As with angiogenesis, inflammation acts as a trigger for microenvironmental change and ECM remodelling which can drive proliferation as discussed previously.

### Pro-metastatic ECM and stroma

Just as stromal and ECM composition can be engineered to promote quiescence; they can also enable cell cycle re-entry. Aging, scarring or fibrosis can trigger increased deposition of Collagen-1 in the lungs and breast, both of which lead to matrix stiffening and increased FAK/RAS/ERK signals that drive proliferation [128–130]. COL1 also binds the Discoidin domain receptor (DDR1) causing further JAK/STAT3 signalling capable of activating SOX2 and MYC expression to increase proliferation [95, 131]. Matrix stiffness was shown to activate quiescent hepatocellular carcinoma cells (HCCs) via increased TGFb1 signalling that drives CyclinD1/3 expression [70]. Fn-rich matrices have been shown to induce an ERK^high^/p38^low^ signalling ratio leading to high CDK activity. In this case, tight Fn bundles inhibit the activation of p38, while urokinase plasminogen activator receptor (uPAR) binds the a5b1-integrin and drives high ERK activation [93, 132]. In some instances, DTCs can manipulate their own niche for quiescence exit, such as breast cancer cells, which deposit TNC once metastasised to the lung. TNC deposition and binding promotes Wnt pathway signalling, and subsequently increases CDK activity via expression of MYC and CyclinD [133]. Breast cancer cells have also been shown secrete MMP9, which encourages exit from quiescence via angiogenesis or migration to new proliferative environments [134]. Similar research has revealed neutrophil extracellular traps (NETs) which promote quiescent to proliferation transitions by secretion of MMP-9. MMP-9 mediates the cleavage of laminin-111 and reveals integrin α3β1 activating epitopes that lead to increased FAK/ERK signalling [135].

We previously touched on how the expression of Wnt5a, an activator of quiescence for breast and prostate cancer cells, declines with age and possibly contributes to relapse in older age [36, 136]. Fane et al. [137] have since confirmed Wnt5a as a driver of dormant melanoma metastases in the lung. Using a series of in vitro and in vivo experiments they highlight how age-induced reprogramming of stromal fibroblasts in the lung increased their expression of sFRP1 leading DTCs to break from quiescence. sFRP1 antagonises Wnt5a leading to a loss of the dormancy phenotype and promoting metastatic outgrowth in mouse models. The examples above neatly demonstrate how tissues can evolve over time to support metastatic outgrowth rather than dormancy.

## Discussion

### Converging signals at the metastatic niche

**Fig. 2:**
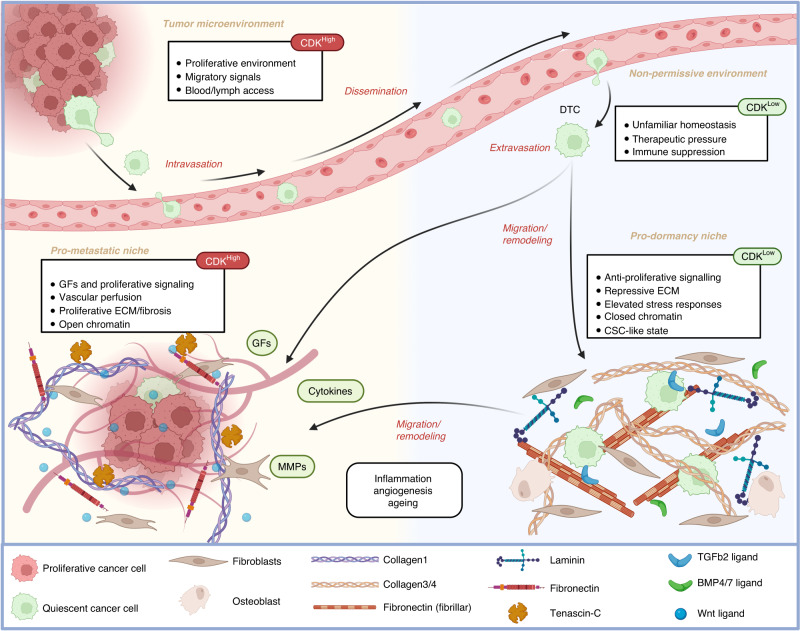
Cellular and acellular factors effecting DTC quiescence and outgrowth. Metastatic tumour cells enter quiescence prior to or during dissemination in the blood or lymph and can extravasate into new tissues. New tissues present unfamiliar homeostatic mechanisms which can continue to suppress growth for a time. Reciprocal signalling between DTCs and their environment drive migration or niche remodelling to generate a pro-metastatic or pro-dormancy niche. These niches effect quiescence/proliferation decisions by influencing the activity of CDKs in the cell.

Here, we have explored how DTC quiescence can be induced, maintained, and exited, through signals which ultimately converge on the activity of CDKs through common CDK regulators (Fig. 1). Figure 2 depicts how CDK activity evolves over time as cells disseminate, extravasate, migrate and remodel their environments to establish 'pro-dormancy’ or ‘pro-metastatic’ niches. While this is an effective model of metastasis and tumour dormancy, it is an oversimplification to suggest microenvironments are exclusively proliferative or quiescent in nature. In reality, DTCs frequently integrate opposing signals from the same niche, which combine to modulate the threshold of CDK activity and thus determine if cells will proliferate or enter quiescence.

We have seen how the BM provides an effective ‘pro-dormancy’ niche through myriad secreted factors, cell-cell interactions, and ECM deposition [41, 42, 138]. However, the BM is also a site of regenerative proliferation and differentiation of bone and immune cells in response to infection or injury, and as a result is rich in growth factors such as VEGF, IGFs, FGFs and BMPs [139–142]. BM-derived fibroblasts can drive HSC proliferation by secreting FGF-2 and -4 leading to ERK and PI3K activation (Fig. 1b) [143]. Despite this, the numerous strong quiescent signals in this BM niche mean the net effect on DTCs remains suppressive. In other organs the net difference between opposing signals may be much smaller, meaning quiescence is more short-lived in these niches. These dynamic niches are also subject to change following reciprocal signalling with DTCs, predictable evolution with age, and stochastic changes brought about by inflammation, scarring and repair. We have seen how quiescence in the perivascular niche of the lungs, supported by secreted Wnt5a and BMP signalling [36, 52, 136], can be counteracted by Wnt5a inhibition from aging stromal cell secretion of sFRP1, or Collagen1 deposition following scarring or ageing [129, 130].

The plasticity and heterogeneity of metastatic cancers and their niches makes a thorough comparison of dormancy mechanisms challenging, though common pathways are emerging as the literature expands. By clarifying the interplay of quiescent and proliferative signals at each site we can better understand the events leading to dormancy and outgrowth, which could lead to novel therapeutic opportunities in the future.

### Clinical opportunities for tumour dormancy

**Fig. 3:**
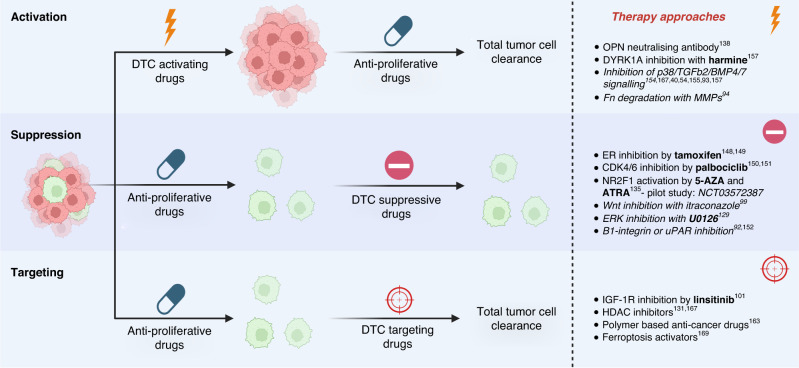
Schematic showing the three dormancy treatment methods with examples. Quiescent cancer cells can be tackled using suppressive, activating, or specific targeting drugs. These can be applied in combination with typically anti-proliferative cancer drugs to reduce the number of DTCs and repress cancer outgrowth.

Since conventional therapies target highly proliferative cells, quiescent DTCs possess an elevated tolerance to most treatments, which can be enforced by elevated stress response pathways and repression of apoptotic pathways (as reviewed here [144, 145]). While there are currently very few drugs specifically targeting quiescent cells, as our understanding of cellular quiescence and tumour dormancy improves, an increasing number of drugs are being co-opted to tackle quiescent DTCs, with many more in pre-clinical development [146–148]. Though many types of drugs are emerging, their applications fall within three techniques for treating tumour dormancy: ‘Suppression’, ‘Activation’ or ‘Targeting’ (Fig. 3).

Suppression describes a method by which relapse is prevented either by consolidation of pro-quiescence signals and the quiescent niche, or by inhibition of activating proliferative signals (Fig. 3). Inhibiting proliferative signals in ER+ breast cancer with the ER antagonist Tamoxifen is an established primary treatment and is now given up to 10 years after diagnosis to reduce relapse and improve patient survival [149, 150]. A similar application has now been approved for the CDK4/6 inhibitor Palbociclib, which is applied to metastatic ER+, HER2- breast cancer to keep residual DTCs quiescent [151, 152]. A new pilot study (*NCT03572387)* is assessing the use of retinoic acid (RA) 5-AZA and ATRA in prostate cancer patients to prolong dormancy by inducing a quiescent expression signature through activation of NR2F1 [146]. Alongside pre-approved drugs, many pre-clinical targets have been identified as effective DTC suppressors. Direct inhibition of proliferative Wnt signals with Wnt5a or DKK1 [36, 100], or ERK signalling with U0126 [130], as well as repressing ERK activators uPAR and B1-integin [93, 153], have been shown to prevent outgrowth in pre-clinical models. Alternatively, upregulation of pro-dormancy signals like p38 [31, 41, 55, 154], TGFb2 [55, 155] and BMP4/7 [86, 156] have all been shown to reduce DTC proliferation in cell and animal models. Suppression has been shown to be effective in some instances but carries challenges regarding prolonged treatment toxicity, high economic cost, and possible selection for therapy resistant relapses.

Activation aims to re-establish DTC susceptibility to standard anti-proliferative therapies, to reduce or eradicate the quiescent cell population through primary treatment (Fig. 3), typically by inhibiting pro-dormancy factors. Evidence from Boyerinas et al. [138] showed how Cytarabine treatment of leukaemic mice could be supplemented by OPN neutralisation to reduce the number of DTCs in the BM niche. Results from these preclinical models found that no residual disease was detectable, and relapse was significantly reduced [138]. In similar studies inhibiting DYRK1A [157] with Harmine improved the efficacy of Imatinib in clearing gastrointestinal cancer in mice [158], while DYRK1B inhibition combined with Gemcitabine was shown to improve pancreatic cancer cell killing in vitro [159]. DYKR1 is a kinase involved in the assembly and activation of the cell cycle inhibiting DREAM (dimerization partner, RB-like, E2F and multi-vulval class B) complex [160, 161]. DYKR1 and the DREAM complex are known to contribute to ovarian cancer quiescence, with in vitro evidence showing DYRK1 inhibition causes cancerous ovarian spheroids to lose viability and cells to exit quiescence [162]. Though there is evidence that activation can improve fractional killing in the first instance, few activation methods have reached clinical trials as they carry significant risk to recently diagnosed patients. This is because activation may exacerbate malignant phenotypes and establish a more aggressive cancer in patients.

Targeting offers the lowest risk to patients by exploiting highly specific pathways to eliminate DTCs. Some promising examples have been identified in pre-clinical studies (Fig. 3), but for direct targeting of quiescent cells to be a feasible method, it will require huge progress in our understanding of different quiescent cell populations. The most likely solution will come from combinatorial treatment based on specific information regarding the cancer subtype and genetic profile. Clinical studies have begun to explore Palbociclib in combination with various other adjuvant therapies (NCT04841148) targeting autophagy and the programmed cell death checkpoint, to optimise DTC clearance. Indeed, the use of clinically approved HDAC inhibitors to open chromatin and reawaken quiescent cells is viable but is only most effective when used in conjunction with other chemotherapies [146, 163]. By shifting plastic cancer cells to more vulnerable states and eliminating their survival mechanisms, we can reduce residual disease during the first round of treatment [164].

Despite significant interest in recent years, our understanding of the role of cellular quiescence in tumour dormancy remains far from complete, particularly considering the heterogeneity of cancer types and their metastatic sites. Though good progress has been made in existing models, the lack of diversity in the models used for investigating tumour dormancy is likely limiting our understanding of these heterogeneous cells. As well as increasing diversity, effort must be made to create preclinical models that can better simulate the kinetics of residual disease, to view the role of metastasis, niche modification, and cell-cell interactions simultaneously.
